# Feedback loops and circular causality: a grammar to restore harmony to physiological systems

**DOI:** 10.31744/einstein_journal/2026RW2240

**Published:** 2026-09-15

**Authors:** Felipe Coelho Argolo

**Affiliations:** 1 Hospital das Clínicas Faculdade de Medicina Universidade de São Paulo São Paulo SP Brazil Instituto de Psiquiatria, Hospital das Clínicas, Faculdade de Medicina, Universidade de São Paulo, São Paulo, SP, Brazil.

**Keywords:** Causality, Physiological feedback, Homeostasis

## Abstract

Causal inference and its graphical representation using directed acyclic graphs provide invaluable tools for calculating effects and handling confounding in medicine. However, since biological systems involve complex dynamics characterized by numerous feedback loops, practitioners need to abstract out the inner workings of cycles in order to reason with a causal framework. This text presents a step beyond linear causality, toward a language that can adequately capture the circular complexities underlying human health. The most fundamental homeostatic mechanisms are inherently cyclic, including sleep-wake states, hormonal regulation, hemodynamics, respiration, blood cell production, eating, and digesting. In fact, numerous medical interventions aim to restore harmonious activity within such systems, reversing dysfunctional states by appropriately modifying certain elements. These include sleep hygiene, synchronized cardioversion, mechanical ventilation, glycemic periodic control with insulin. This study describes medical contexts using cyclic graphs, covering interventions such as control of hormonal cycles in reproductive medicine. Health practitioners can benefit from this enriched perspective, which parsimoniously describes the fundamental behavior of disease processes.

## INTRODUCTION

Over the last decades, medicine has embraced graphs and networks as basic abstractions for describing complex relationships among interconnected elements. Their remarkable versatility allows researchers to model and analyze biological organization at vastly different scales using the common mathematical language of graph theory. In this representation, system components are modeled as nodes (or vertices) and their pairwise interactions as edges.^(1)^

Network models have yielded significant insights across the full spectrum of biological organization. At the molecular level, graph theory provides a framework for modeling the intricate machinery of the cell. By representing proteins as nodes and their physical binding interactions as edges, researchers can construct protein-protein interaction networks to identify “disease modules” – defined regions of a network in which components are collectively implicated in a specific pathology.^(2)^ This approach has been instrumental in elucidating complex mechanisms, such as the coagulation cascade. At the organ level, graph theory has become central to modern fields such as neuroscience. The brain is readily conceptualized as a network in which nodes can represent individual neurons or entire cortical regions, and edges can denote structural connections (axonal pathways) or functional connectivity, defined as statistical dependencies between activity time series.^(3)^ Such formulations describe brain architecture, development, and the network-level disruptions underlying disorders such as Alzheimer’s disease and depression.^(4)^

Various fields related to medicine, from neuroscience to molecular biology, have developed bespoke causal concepts and analytical toolkits based on directed acyclic graphs (DAGs).^(5)^ As implied by their name, DAGs do not contain cycles, requiring workarounds to describe structures such as feedback loops. This study introduces concepts that aim to fill this gap, providing a language for describing cyclical biological networks using directed cyclic graphs (DCGs). Known interventions, such as the control of hormonal cycles, can be simplified and better understood through abstractions that parsimoniously represent their fundamental behavior. The proposed framework represents a necessary step beyond linear causality toward a language that can adequately capture the complexity of processes underlying disease. In addition to its heuristic and philosophical implications, we present a data-driven framework for identifying networks and their underlying dynamics.

### On disease causes and directed cyclic graphs

A coherent mathematical and conceptual framework for operationalizing causal reasoning with graphs was established by Judea Pearl. It has had a profound effect on epidemiology and clinical research, offering a powerful toolkit for addressing problems that were once considered intractable.^(6)^

The centerpiece of the theory comprises three layers of causation.

1. Association (Seeing). This is the level of classical statistics, concerned with observational relationships and questions such as “What is the probability of a disease given a certain symptom?” It is represented by the conditional probability *P(y|x)*.
2. Intervention (Doing). This level addresses questions about the effects of active manipulation, including the question, ‘What would happen to the disease rate if we administered a drug? It is represented by the do-calculus expression *P(y|do(x))*. It requires more than just observational data; it requires a causal model or a controlled experiment in which the intervention is physically implemented.
3. Counterfactuals (Imagining). This level addresses questions about events that did not occur, such as, “Would a patient who died after taking the drug have survived if they had not taken it?”

The primary tool for operationalizing this framework is the DAG. A DAG is a visual representation of the assumed causal structure of a system, in which variables are nodes and causal influences are directed arrows. The principal utility of DAGs in medicine has been to provide a formal, graphical method for identifying and controlling confounding in observational studies. By mapping assumed causal pathways between an exposure and an outcome, researchers can identify “backdoor paths” – non-causal associations created by common causes – and determine the minimal set of covariates that must be adjusted for in a statistical model to block these paths and isolate the true causal effect.^(7)^

The visual and intuitive nature of DAGs serves as a form of “cognitive prosthesis”, allowing researchers to make their causal assumptions explicit, share them with colleagues, and subject them to debate in a structured manner. However, the mathematical power and conceptual clarity of DAGs derive from a single, foundational constraint: the “A” in DAG stands for acyclic. This means that it is impossible to start at any node and follow a sequence of directed arrows back to the same node.^(8)^

In contrast, life is a dynamically stable process maintained by a constant interplay of feedback loops. Physiological homeostasis, the bedrock of health, is governed by numerous feedback cycles, from the regulation of body temperature to the intricate hormonal cascades of the endocrine system. The hypothalamic-pituitary-adrenal (HPA) axis, for instance, is a canonical example in which cortisol feeds back to inhibit its own production by the hypothalamus and pituitary gland. ^(9)^

This structural limitation forces researchers using DAGs to adopt workarounds. One approach is abstracting out the cycles: an entire cyclic process is “black-boxed” into a single node, compressing its internal dynamics and resulting in a loss of information.^(10)^ Another approach is to “unwrap” the graph over time, creating a linear chain of variables indexed by time steps (e.g., X_t_→Y_t+1_→X_t+2_). This approach can become infinitely complex and may lose the parsimonious representation of the underlying recursive cycle.^(11)^

The acyclicity requirement imposes a linear, sequential, and non-reciprocal narrative on the world, a kind of philosophical straitjacket.^(12)^ It reflects a deeper epistemological problem the imposition of a linear, sequential narrative on biological processes that are fundamentally defined by coupled dynamics and strange feedback loops.

This paper argues that moving beyond this limitation requires not just a new tool, but a new grammar for describing life – one that aligns with the holistic and self-regulating nature of living systems. This axiom of acyclicity, while powerful for identifying confounding in static scenarios, is fundamentally at odds with the principle of homeostasis, which can be better described through circular causality. In such systems, the neat separation between cause and effect dissolves into a process of mutual regulation.

### A cyclic grammar of graphs: cybernetics

The formal study of systems with circular causality began in the 1940s and 1950s under the banner of cybernetics, a field forged in the interdisciplinary Macy Conferences.^(13)^ Wiener,^(14)^ a central figure in cybernetics, defined it as the science of “control and communication in the animal and the machine”, with the concept of the feedback loop as its foundational element. Wiener and his contemporaries, such as John von Neumann and Ross Ashby, sought to understand how complex systems – whether biological organisms or advanced machines – could maintain stability, adapt, and pursue goals in changing environments. Their answer lay in circular causal processes in which the output of an action is fed back as input to guide future actions.^(14)^

A crucial precursor to this work was the 1943 paper by McCulloch et al.,“A Logical Calculus of the Ideas Immanent in Nervous Activity”^.(15)^ They demonstrated that networks of simple, neuron-like elements could perform complex logical computations. Importantly, their models explicitly included cycles, showing how circular pathways were essential for functions such as memory and reverberating activity. This work laid the groundwork for understanding the brain not as a linear processor, but as a dynamic, recurrent system.

This cybernetic tradition, which embraced circularity to model dynamic, goal-directed behavior, offers a powerful alternative to the static, observational focus of the DAG framework. A DCG – a directed graph that permits cycles – is the natural mathematical representation of this perspective. It allows for the direct and intuitive modeling of the feedback loops that McCulloch, Wiener, and Ashby identified as the very basis of autonomous, adaptive systems.

### Homeostasis as harmony: the physiological primacy of cycles

A cyclic grammar is at the core of the organizing principle of physiology: homeostasis. Homeostasis is the self-regulating process by which living systems maintain a stable internal environment – the “milieu intérieur” – despite continuous external changes. The term itself, derived from the Greek *homoios* (similar) and *stasis* (standing still), captures this idea of dynamic stability. This state of equilibrium is necessary for optimal functioning and is maintained by a vast network of regulatory mechanisms, most of which involve feedback loops.^(16)^

A compelling argument can be made that, given our evolutionary roots, the most fundamental life-sustaining mechanisms are inherently cyclic. Life is not a linear progression but a series of nested rhythms and cycles that maintain a dynamically stable state. These include:

Circadian Rhythms: The sleep-wake cycle governs countless physiological processes, generally synchronized with light availability and regulated by CLOCK/BMAL1 genes.^(17)^

Hormonal Regulation: The pulsatile release of hormones and the cyclical nature of endocrine axes like the menstrual cycle and the HPA (hypothalamus - pituitary - adrenal) axis.^(9)^

Hemodynamics: The cardiac cycle of systole and diastole ensures blood circulation.^(18)^

Respiration: The cycle of inhalation and exhalation.^(19)^

Metabolism: The cycles of eating, digestion, and nutrient utilization, including the tight regulation of blood glucose.^(20)^

This perspective allows for a powerful formal representation of the biological state of homeostasis, providing a modern, systems-level view that resonates deeply with ancient concepts of health as “harmony” or “balance” between competing elements, such as the four humors described by Hippocrates.

Consequently, disease can be understood as a state of “disharmony”, involving the disruption of these vital homeostatic cycles.^(21)^

This systems-level view of health as a form of dynamic stability finds its most rigorous philosophical expression in the 20th-century work of Canguilhem, who argued against the prevailing view of disease as a mere quantitative deviation from a statistical average. Instead, he defined health as biological normativity: the capacity of a living organism to overcome challenges and tolerate environmental variations by instituting new norms of functioning. Disease, in this framework, is not a lack of norms but a reduction to a single, restricted norm, reflecting an inability to adapt that renders the organism fragile.^(22)^

Pathology often manifests as faulty behavior in composite systems that depend on balanced feedback. For instance, a stable negative feedback loop can devolve into a runaway pathological positive feedback loop, as seen in cytokine storms.^(23,24)^ A necessary negative feedback circuit can be disrupted, leading to unchecked activity, as occurs in many autoimmune diseases. Alternatively, a dynamic cycle can become locked in a pathological steady state, such as when chronic stress leads to a persistently overactive HPA axis.^(25)^

From this viewpoint, the ultimate goal of many medical interventions is to restore this harmonious cyclic activity. Therapy is reframed from “fixing a broken part to returning a complex, dynamic system to its natural homeostatic dynamics”. At a fundamental level, the restoration of physiological harmony unifies disparate medical fields and interventions, such as sleep hygiene (restoring a circadian cycle), synchronized cardioversion (resetting a cardiac cycle), and hormonal therapy (modulating an endocrine cycle) table 1.

**Table 1:** Cyclic physiological systems, related medical conditions, interventions, and mechanisms of action

Cyclic System	Pathological state (Disharmony)	Intervention	Mechanism of action
Glycemic control	Hyperglycemia (disrupted glucose-insulin loop)	Insulin therapy	Manually replaces the missing negative feedback signal to restore glucose homeostasis
Reproductive system (HPO Axis)	Anovulation / Irregular cycles	Hormonal contraception	Exogenously imposes a stable hormonal state, overriding and suppressing the pathological endogenous cycle
Cardiac conduction	Reentrant tachyarrhythmia	Synchronized cardioversion	Delivers a timed electrical pulse to depolarize the entire myocardium, terminating the pathological circuit and allowing the natural pacemaker to resume control
Respiratory system	Respiratory failure	Adaptive mechanical ventilation	Creates a closed-loop, patient-ventilator system that synchronizes ventilatory support with the patient’s own respiratory drive

### Clinical applications: modeling and restoring harmonic systems

The practical utility of this cyclic grammar is best demonstrated through its application to concrete clinical problems. By modeling physiological systems as DCGs, we can better understand their pathological states and frame interventions as precise acts of restoring cyclic harmony.

### Metabolic regulation: the glucose-insulin loop in *diabetes mellitus*

The regulation of blood glucose is a quintessential example of a negative feedback loop essential to homeostasis. After a meal, rising blood glucose levels stimulates pancreatic beta-cells to release insulin. Insulin promotes glucose uptake by cells, lowering blood glucose levels. As glucose levels fall, insulin secretion decreases, and the pancreas releases glucagon, which stimulates the liver to release stored glucose, thereby raising blood glucose levels. This interaction can be elegantly modeled as a DCG, with the core components – glucose, insulin, and glucagon – forming a regulatory cycle.^(26,27)^

Pathology in this system can result directly from disruption of the regulatory cycle. In type 1 diabetes, the insulin- secreting node, represented by the beta cells, is destroyed by an autoimmune process.

The feedback loop is physically severed, as the control center can no longer produce and release the necessary signal. In type 2 diabetes, the pancreas produces insulin, but the effector nodes, including muscle, liver, and adipose cells become resistant to its signal. The loop’s communication pathway is disrupted, rendering the feedback ineffective.

Medical intervention for diabetes can be understood as the creation of an *artificial, external feedback loop* to replace the disrupted endogenous one. The patient, using a glucometer to measure blood glucose levels (the receptor) and administering calculated insulin doses (the effector), becomes the new control center. This conscious, managed process forms a new DCG that aims to restore the homeostatic harmony that the body can no longer maintain on its own.^(26)^

### The rhythms of life: feedback control in reproductive medicine

The female menstrual cycle is governed by the hypothalamic-pituitary-ovarian (HPO) axis, a complex regulatory system that employs both negative and positive feedback to regulate reproductive function. Throughout most of the cycle, estrogen and progesterone exert negative feedback on the hypothalamus and pituitary gland, suppressing the release of gonadotropin-releasing hormone (GnRH), follicle-stimulating hormone (FSH), and Luteinizing hormone (LH). However, at midcycle, a sustained high level of estrogen paradoxically switches the system to positive feedback, causing a massive LH surge that triggers ovulation. This dynamic interplay of feedback mechanisms creates the precise hormonal rhythm required for fertility.^(28,29)^

Pathological conditions such as polycystic ovary syndrome (PCOS) or hypothalamic amenorrhea can be understood as disruptions in this delicate cyclic timing, amplitude, or frequency. Interventions in reproductive medicine are therefore best viewed not as simple agonists or antagonists but as powerful tools for modulating the parameters of the cycle. Oral contraceptives, for example, introduce exogenous hormones that establish a stable state of negative feedback, suppressing the endogenous cycle and preventing ovulation. Conversely, fertility treatments aim to amplify or trigger specific phases of the cycle to induce follicular development and ovulation. These therapies are fundamentally about controlling or restoring a biological rhythm.

### Restoring resonance: interventions that harmonize cyclic activity

Some medical interventions directly reset or entrain a pathological biological rhythm.

Synchronized Cardioversion: The heart’s electrical conduction system is a biological oscillator. Tachyarrhythmias, such as supraventricular tachycardia (SVT) or ventricular tachycardia (VT) with a pulse, are pathological, high-frequency cycles often caused by a re-entrant electrical circuit. Synchronized cardioversion is a procedure that delivers a low-energy electrical shock to terminate these arrhythmias. Importantly, the shock is *synchronized* with the R-wave of the QRS complex on the electrocardiogram. This precise timing is essential to avoid delivering the shock during the vulnerable repolarization period (the T-wave), which could induce lethal ventricular fibrillation. The shock causes a critical mass of cardiac myocytes to depolarize simultaneously, interrupting the pathological re-entrant circuit. This “resets” the entire myocardium, allowing the heart’s natural pacemaker (the sinus node) to regain control and re-establish a normal, harmonic rhythm. This is a direct, phase-specific intervention designed to interrupt one cycle and restore another.^(30)^

Mechanical Ventilation: In critical care, the cardiorespiratory system is a network of coupled oscillators. Modern mechanical ventilators are not simply air pumps; they are sophisticated closed-loop feedback systems designed to work with the patient’s physiology. Modes such as proportional assist ventilation (PAV) and adaptive support ventilation (ASV) continuously monitor the patient’s respiratory rate, volume, and effort. They then adjust the delivered support on a breath-by-breath basis to “harmonize patient-ventilator interaction”. The goal is not to override the patient’s respiratory drive but to create a synchronous, coupled system between the patient and the machine. This process of physiological entrainment restores the fundamental rhythm of breathing while reducing the patient’s work of breathing, thereby facilitating recovery.^(31)^

### Decomposing complexity: a grammar of fibers and cycles

The complexity of biological systems, such as the cardiac cycle described above, arises from dense networks of feedback loops that are difficult to untangle using linear causal models. To rigorously analyze the structure of these systems and concepts such as network fibers, we can benefit from formal representations. To this end, two canonical examples of physiological feedback are analyzed with computational models: the cardiac pressure-volume loop and the human menstrual cycle. Simulations are used to show how data-driven algorithms can capture the dynamics of biological systems using raw observations such as multivariate time series of hormones or hemodynamic parameters.

Our first simulation implements a model of the heart’s mechanical cycle, generating the characteristic sawtooth pattern of repeated heartbeats. The second implements a well-established model of the human menstrual cycle, capturing the complex hormonal interplay within the ovary. This model successfully produces a stable, repeating cycle of approximately 30 days, mirroring the oscillatory nature of the reproductive system. The time-series data from these simulations provide rich datasets from which the systems’ internal feedback structures can be inferred (Figure 1).

**Figure 1:**
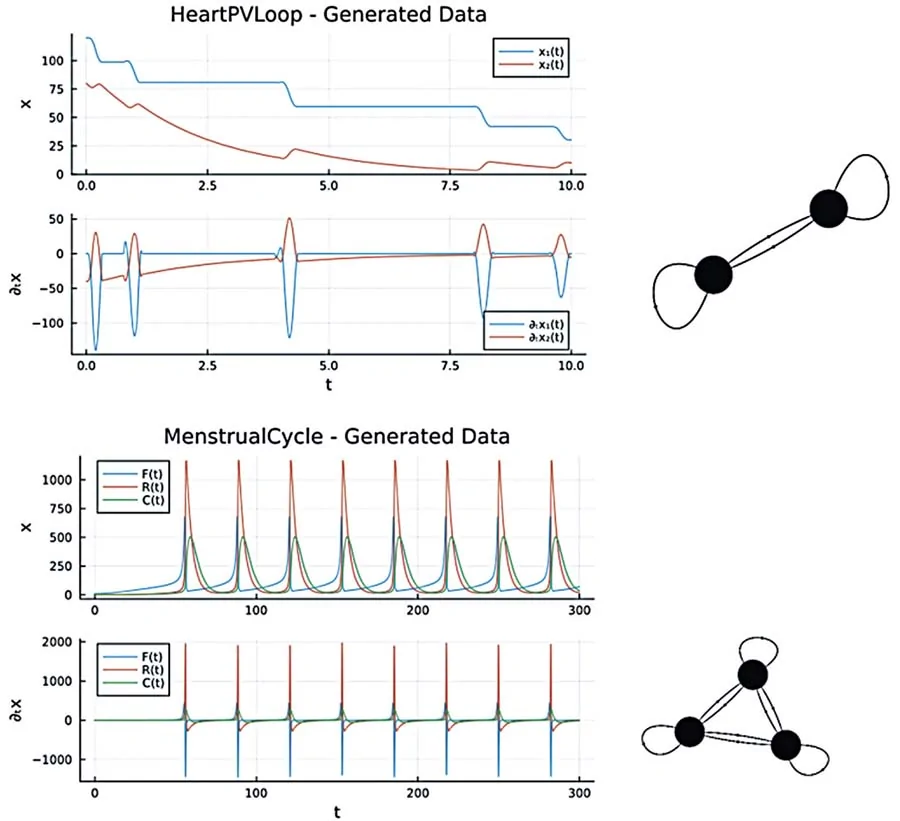
Time-series obtained from generative models and the corresponding cyclic graphs obtained after system identification. See Table 1S, Supplementary Material for details of the implementation and equations

Using a data-driven system identification algorithm, we can automatically reverse- engineer the underlying interaction networks from these simulated data. This process generates a directed graph for each model, which serves as a formal representation of the feedback pathways. These graphs are the precise mathematical objects that can be analyzed, allowing us to formally identify symmetries and coordinated behavior within these complex physiological feedback loops. Both systems (cardiac activity and reproductive hormonal signaling) have already been accurately characterized. However, researchers are still trying to identify the underlying dynamics of several networks, such as the molecular interactions among interleukins in sepsis.^(32,33)^

## DISCUSSION

### Toward a new therapeutic horizon

The adoption of a grammar that is native to the cyclical and symmetric realities of physiology is not an end in itself. A language that can articulate elements cyclically allows us to generate new questions and reveal novel points of leverage for therapeutic intervention.

It is crucial to emphasize that this cyclic grammar is not intended to replace the DAG framework. DAGs remain essential and appropriate tools for their intended purpose: identifying confounding and estimating causal effects in situations in which a linear, acyclic causal structure can be reasonably assumed.^(6)^ The cyclic framework provides the appropriate tool for a different, and arguably more fundamental, class of biological problems involving dynamic feedback and homeostatic control.

The treatment strategies discussed, such as synchronized cardioversion, are inherently state-dependent. In this type of intervention, the timing and nature of the intervention depend on the system’s trajectory. Previous studies have addressed nuances of medical interventions that account for temporal patterns,^(34-36)^ generating further discussion in the literature.^(37)^ This shift in perspective creates a fertile environment for designing more sophisticated therapeutic strategies. It provides a formal, first-principles foundation for emerging fields such as chronomedicine, which seeks to time drug administration to align with the body’s circadian rhythms. It also explains the known benefits of pulsatile hormone replacement, in which the timing of delivery is as important as the dose. A cyclic network model that maps these feedback topologies is critical for the designing of rational therapies.

## CONCLUSION

### Beyond causality, toward control

The causal inference framework based on directed acyclic graphs has been a monumental achievement for medical science, providing essential tools for navigating the complexities of confounding in observational studies. However, its foundational axiom of acyclicity represents a philosophical and practical barrier to understanding the deeply cyclical and redundant nature of biological systems. The linear grammar of directed acyclic graphs, while powerful for its intended purpose, is insufficient for parsimoniously describing the rich, dynamic feedback that constitutes life itself.

This paper has argued for an expansion of our descriptive language, through the adoption of a grammar of cycles and symmetries that is native to the realities of physiology. This framework provides the conceptual scaffolding needed to formally describe many of our most effective therapeutic strategies, from harmonizing hormonal cycles to resonance-based interventions such as cardiac pacing.

This framework represents a necessary step beyond a purely linear view of causality, toward a grammar that can adequately capture the complexity of the processes underlying disease. It encourages a shift in focus from a static search for “causes” to a dynamic understanding of control. By embracing this perspective, we can begin to design interventions that work with the complex, rhythmic nature of the patient, gently restoring balance and harmony rather than simply acting upon the patient.

## SUPPLEMENTARY MATERIAL

Supplementary Material

## References

[1] Ivanov PC, Liu KK, Bartsch RP (2016). Focus on the emerging new fields of Network Physiology and Network Medicine. New J Phys.

[2] Silverman EK, Schmidt HH, Anastasiadou E, Altucci L, Angelini M, Badimon L (2020). Molecular networks in Network Medicine: development and applications. Wiley Interdiscip Rev Syst Biol Med.

[3] Farahani FV, Karwowski W, Lighthall NR (2019). Application of Graph Theory for Identifying Connectivity Patterns in Human Brain Networks: A Systematic Review. Front Neurosci.

[4] Zheng K, Wang H, Li J, Yan B, Liu J, Xi Y (2019). Structural networks analysis for depression combined with graph theory and the properties of fiber tracts via diffusion tensor imaging. Neurosci Lett.

[5] Borger MJ, Ramesh A, Let's DA (2025). Let's DAG in: how directed acyclic graphs can help behavioural ecology be more transparent. Proc Biol Sci.

[6] Pearl J (2009). Causality.

[7] Tennant PW, Murray EJ, Arnold KF, Berrie L, Fox MP, Gadd SC (2021). Use of directed acyclic graphs (DAGs) to identify confounders in applied health research: review and recommendations. Int J Epidemiol.

[8] Digitale JC, Martin JN, Glymour MM (2022). Tutorial on directed acyclic graphs. J Clin Epidemiol.

[9] Packard AE, Egan AE, Ulrich-Lai YM (2016). HPA axis interactions with behavioral systems. Compr Physiol.

[10] Dawid AP (2010). Beware of the DAG!. Causality: objectives and assessment.

[11] Hernan M, James M (2023). Causal inference: what if.

[12] Frisch M (2022). Causation in Physics.

[13] Von Foerster H (1952). Cybernetics; circular causal and feedback mechanisms in biological and social systems.

[14] Wiener N (2019). Cybernetics or Control and Communication in the Animal and the Machine.

[15] McCulloch WS, Pitts W (1943). A logical calculus of the ideas immanent in nervous activity. Bull Math Biophys.

[16] Billman GE (2020). Homeostasis: The Underappreciated and Far Too Often Ignored Central Organizing Principle of Physiology. Front Physiol.

[17] Laposky A, Easton A, Dugovic C, Walisser J, Bradfield C, Turek F (2005). Deletion of the mammalian circadian clock gene BMAL1/Mop3 alters baseline sleep architecture and the response to sleep deprivation. Sleep.

[18] Middeke M (2016). The pioneer in hemodynamics and pulse-wave analysis, Otto Frank. J Am Soc Hypertens.

[19] Jablonski I (2013). Modern methods for the description of complex couplings in the neurophysiology of respiration. IEEE Sens J.

[20] Merrins MJ, Corkey BE, Kibbey RG, Prentki M (2022). Metabolic cycles and signals for insulin secretion. Cell Metab.

[21] Bujalkova M, Straka S, Jureckova A (2001). Hippocrates' humoral pathology in nowaday's reflections. Bratisl Lek Listy.

[22] Canguilhem G (2012). On the Normal and the Pathological.

[23] Yiu HH, Graham AL, Stengel RF (2012). Dynamics of a cytokine storm. PLoS One.

[24] Kareva I, Berezovskaya F, Karev G (2022). Mathematical model of a cytokine storm. bioRxiv.

[25] Iob E, Kirschbaum C, Steptoe A (2020). Persistent depressive symptoms, HPA-axis hyperactivity, and inflammation: the role of cognitive-affective and somatic symptoms. Mol Psychiatry.

[26] Bich L, Mossio M, Soto AM (2020). Glycemia Regulation: From Feedback Loops to Organizational Closure. Front Physiol.

[27] Röder PV, Wu B, Liu Y, Han W (2016). Pancreatic regulation of glucose homeostasis. Exp Mol Med.

[28] Kauffman AS (2022). Neuroendocrine mechanisms underlying estrogen positive feedback and the LH surge. Front Neurosci.

[29] Chen CY, Ward JP (2014). A mathematical model for the human menstrual cycle. Math Med Biol.

[30] Goyal A, Singh B, Chhabra L, Singhal M (2023). Synchronized Electrical Cardioversion.

[31] Branson RD (2000). Techniques for automated feedback control of mechanical ventilation. Semin Respir Crit Care Med.

[32] Kim KS, Jekarl DW, Yoo J, Lee S, Kim M, Kim Y (2021). Immune gene expression networks in sepsis: A network biology approach. PLoS One.

[33] Wu D, Wang L, Hong D, Zheng C, Zeng Y, Ma H (2022). Interleukin 35 contributes to immunosuppression by regulating inflammatory cytokines and T cell populations in the acute phase of sepsis. Clin Immunol.

[34] Argolo FC, Gadelha A, Pan PM, Bressan RA (2020). Time-driven interventions for affective disorders: on resonance and the Oberth effect. Trends Psychiatry Psychother.

[35] Argolo FC, Garcia-Dias R, Pan PM, Gadelha A, de Castro Quarantini L, Bressan R (2020). Timing in psychiatry: exploring resonance in biological systems for treatment of mental disorders. OSF Preprints.

[36] Argolo F. C. (2023). Six rationales for medical treatment inspired in the study of dynamical systems. Octopus.

[37] Sanches M (2021). Time-driven interventions for affective disorders and resistance to treatment. Trends Psychiatry Psychother.

