# Supplementary material for: Feedback loops and circular causality: a grammar to restore harmony to physiological systems
Source: Einstein (Sao Paulo). 2026 Sep 15;24(spe1):eRW2240. doi: 10.31744/einstein_journal/2026RW2240 (PMC13689974; doi:10.31744/einstein_journal/2026RW2240)
Supplement: Supplementary file 1 [file 2317-6385-eins-24-nspe1-eRW2240-suppl01.pdf]

SUPPLEMENTARY MATERIAL

Feedback loops and circular causality: a grammar to restore harmony to physiological systems

Felipe Coelho Argolo

DOI: 10.31744/einstein_journal/2026RW2240

Table 1S. Model Formulation and Software Implementation

The simulations and system identification analyses were conducted using the Julia programming language (v1.6 or later) and its Scientific Machine Learning (SciML) ecosystem, particularly the *DifferentialEquations.jl* and *DataDrivenDiffEq.jl* packages.

Full code is available upon request.

A.1 Menstrual Cycle Model

The menstrual cycle is modeled as a system of three delay-differential equations (DDEs), adapted from Chen & Ward [29]. The state variables are the masses of the dominant Follicle F(t), the ruptured follicle or Scar R(t), and the Corpus Luteum C(t). The dynamics are driven by a complex, quasi-steady-state hormonal feedback mechanism involving Estrogen, Progesterone, GnRH, FSH, and LH, with time delays τ_{FSH} and τ_{LH} representing the pituitary response time.

A.2 Heart Pressure-Volume Loop Model

The cardiac cycle is modeled as a simplified Windkessel-like system of two ordinary differential equations (ODEs). The state variables are the ventricular Volume V(t) and the aortic Pressure P(t). The model is driven by a time-varying elastance function E(t), which simulates the periodic contraction and relaxation of the heart muscle.
